# Pre-administration of turmeric prevents methotrexate-induced liver toxicity and oxidative stress

**DOI:** 10.1186/s12906-015-0773-6

**Published:** 2015-07-22

**Authors:** Adel Rezaei Moghadam, Soheil Tutunchi, Ali Namvaran-Abbas-Abad, Mina Yazdi, Fatemeh Bonyadi, Daryoush Mohajeri, Mohammad Mazani, Hassan Marzban, Marek J. Łos, Saeid Ghavami

**Affiliations:** Faculty of Veterinary Medicine, Tabriz Branch, Islamic Azad University, Tabriz, Iran; Young Researchers and Elite club, Tabriz Branch, Islamic Azad University, Tabriz, Iran, Shiraz, Iran; Faculty of Veterinary Medicine, Tehran University, Tehran, Iran; Faculty of Medicine, Tehran Branch, Islamic Azad University, Tehran, Iran; Department of Pathobiology, Tabriz Branch, Islamic Azad University, Islamic Azad University, Tabriz, Iran; Department of Biochemistry, Ardabil University of Medical Science, Ardabil, Iran; Department of Human Anatomy and Cell Science, College of Medicine, Faculty of Health Sciences, University of Manitoba, Winnipeg, Canada; The Children Hospital Research Institute of Manitoba, College of Medicine, Faculty of Health Sciences, University of Manitoba, Winnipeg, Canada; Department of Clinical and Experimental Medicine (IKE), Division of Cell Biology, and Integrative Regenerative Medicine Center (IGEN), Linköping University, Linköping, Sweden; Department of Pathology, Pomeranian Medical University, Szczecin, Poland; ENT Department, School of Medicine, Medical University of Silesia, Katowice, Poland; Health Policy Research Centre, Shiraz Medical University, Shiraz, Iran

**Keywords:** Lipid peroxides, Total antioxidant status, Antioxidant enzymes, Serum liver biomarkers, Hepatocellular injury, Cell death

## Abstract

**Background:**

Methotrexate (MTX) is an antimetabolite broadly used in treatment of cancer and autoimmune diseases. MTX-induced hepatotoxicity limits its application. We investigated hepatoprotective effects of turmeric in MTX-induced liver toxicity.

**Methods:**

All experiments were performed on male Wistar albino rats that were randomly divided into six groups. Group one received saline orally for 30 days (control group), groups two and three received turmeric extract (100, 200 mg/kg respectively) orally for 30 days, group four received single dose, of MTX IP at day 30, groups five and six received turmeric extract 100 and 200 mg/kg orally respectively for 30 days and single dose of methoterxate IP (20 mg/kg) at day 30. Four days after MTX injection animals were sacrificed and evaluated. Blood ALT and AST (indicators of hepatocyte injury), ALP and bilirubin (markers of biliary function), albumin (reflect liver synthetic function) as well as the plasma TAS concentration (antioxidant defenses) were determined. The cellular antioxidant defense activities were examined in liver tissue samples using SOD, CAT, and GSH-Px for the oxidative stress, and MDA for lipid peroxidation. In addition, liver damage was evaluated histopathologically.

**Results:**

MTX significantly induced liver damage (*P* < 0.05) and decreased its antioxidant capacity, while turmeric was hepatoprotective. Liver tissue microscopic evaluation showed that MTX treatment induced severe centrilobular and periportal degeneration, hyperemia of portal vein, increased artery inflammatory cells infiltration and necrosis, while all of histopathological changes were attenuated by turmeric (200 mg/kg).

**Conclusion:**

Turmeric extract can successfully attenuate MTX-hepatotoxicity. The effect is partly mediated through extract’s antinflammatory activity.

## Background

MTX has is a versatile drug that has been clinically applied in a wide range of diseases including systemic lupus erythematosus, rheumatoid arthritis, psoriasis, and neoplastic diseases [1–4]. The therapeutic applications of MTX is usually limited by its severe hepatotoxicity which causes malabsorption and diarrhea in the patients [5, 6]. The underlying mechanism of MTX-induced hepatotoxicity has not been fully identified, however, several distinct mechanisms have been proposed including cellular antioxidant defense deregulation which cause more oxidative stress-induced damages to the liver cells [7]. Mitochondria are the primary intracellular target for oxidative stress [8], however reactive oxygen metabolites scavenger systems like superoxide dismutase (SOD), glutathione peroxidase (GPx), catalase and glutathione-S-transferrase (GST), continuously remove ROS and maintain mitochondria function in an steady state [9, 10]. MTX is indirectly involved in liver mitochondria damage via depletion of mitochondria enzymatic and non-enzymatic antioxidants machinery [11–13]. MTX administration may also cause the accumulation of reactive oxygen species (ROS), targeting macromolecules and many pathological processes [14, 15]. The first line of defense against the cell damaging effects of oxidative stress for liver is antioxidant defense system which neutralizes the deleterious effects of free radicals [16]. Therefore, the level of ROS in liver tissue may be estimated by the analysis of the antioxidant enzymes activity including superoxide dismutase (SOD), catalase (CAT), and glutathione-S-transferase (GST) [17, 18].

Turmeric (*Curcuma longa* L., zingiberaceae), is a yellow-colored spice derived from the perennial herb, which has been widely used for centuries in traditional therapies for different diseases including cancer, inflammatory disorders, hepatitis and other liver disorders, skin diseases, Alzheimer’s disease, rheumatoid arthitis [19–22]. Besides curcumin, turmeric contains more than 300 other components, including, notably phenolics and terpenoids which may more widely and strongly affect various molecular and biochemical cascades [23, 24]. Turmeric provides protection against hepatic damage induced by free radicals and attenuates hepatic lipid peroxidation [25, 26]. Previously, it has been shown in a short term-trial (five days), that curcumin, a main integrant of turmeric, could alleviate hepatic injury induced by MTX, when applied after MTX treatment [13]. However, Gupta and colleagues concluded that the effect of whole turmeric may markedly differ from curcumin itself [27]. The effect of long term administration of whole turmeric on hepatic injury induced by MTX are yet completely unknown. In the current study, using a rat model, we have investigated the effect of pre-administration of turmeric in MTX-induced liver injury. The observed effects were assessed by measuring the levels of antioxidant enzymes, lipid peroxidation marker, total antioxidant status, as well as biochemical and histopathologic evaluations.

## Methods

### Animals and study protocol

Experiments were performed on male Wistar Albino rats weighing between 220–280 g purchased from Drug Applied Research Center, Tabriz University of Medicine Science. Rats were kept in 12 h light and 12 h dark cycle at the temperature of 21 ± 2 °C, food and water were available ad libitum throughout the study. Investigations using experimental animals were conducted in accordance with the internationally accepted principles for laboratory animal use and care as found in the United States guidelines (United States National Institutes for Health publication # 85–23, revised in 1985) and the Institutional Animal Ethics Committee of the Ardabil University of Medical Sciences approved the protocol (# 90374).

42 rats were randomly divided into six groups, group *I* received normal saline orally for 30 days (control group), group *II* and *III* received oral turmeric extract (100 and 200 mg/kg respectively) by gastric gavages for 30 days, group *IV* received single IP MTX (20 mg/kg) on day 30, group *V* received oral turmeric extract (100 mg/kg) for 30 days and IP single dose of MTX (20 mg/kg) on day 30, and group *VI* received oral turmeric extract (200 mg/kg) for 30 days and IP single dose of MTX (20 mg/kg) on day 30. Four days after MTX injection animals were anesthetized; blood and tissue samples were collected for different experiments. Study protocol is summarized in Table 1.

**Table 1:** Schematic diagram of the study protocol

Groups	Number of rats	Treated protocol	Drugs received	MTX dose	TUR dose
		(For 30 days)	(On 30th day)	(mg/kg/day)	(mg/kg/day)
I	7	Saline	NaCl	-	-
II	7	TUR	NaCl	-	100
III	7	TUR	NaCl	-	200
IV	7	Saline	MTX	20	-
V	7	TUR	MTX	20	100
VI	7	TUR	MTX	20	200

*TUR* Turmeric, *MTX* Methotrexate

### Chemicals

MTX was purchased from EBEWE Pharma Ges, Austria. All kits were provided by Randox® (Cat No. NX2332, Randox laboratories, Ltd, Crumlin, UK). Dimethyl sulfoxide (DMSO), ethylene diamine tetracetic acid (EDTA) 3-(4,5-dimethylthiazol-2-yl), ethanol, ethyl acetate, Tris HCl, 2-thiobarbituric acid (TBA) and other chemicals were obtained from Sigma Chemical Co. (St Louis, MO, USA). All chemicals used were analytical grade. Turmeric (*Curcuma longa* L., Zingiberaceae) was purchased from the local market and identified by the Department of Cultivation and Development of Institute of Medical Plants, Tehran, Iran. The plant was then thoroughly washed, air dried, and grinded to a uniform powder. Turmeric powder (10 g) was mixed in distilled ethanol (100 ml) for 3 h at room temperature and was then centrifuged at 2500 rpm for 15 min at 4 °C. This mixture was three times filtered and then the supernatant was evaporated by employing rotary under reduced pressure at 40 °C. Such prepared turmeric extract was used at two different concentrations: 100 mg/0.2 mL, 200 mg/0.2 mL [28]. The voucher specimen (no. 12215) was deposited at the herbarium of Ardabil University of Medical Science.

### Serum biochemical factors evaluation

Blood samples collected from the retro-orbital plexus were centrifuged at 2500 rpm for 15 min at 30 °C for sera preparation. The sera were then stored at −30 °C and later the following parameters were measured: alanine aminotransferase (ALT), aspartate aminotransferase (AST), alkaline phosphatase (ALP), Albumin (ALB), and total protein (TP). ALT and AST are markers for hepatocyte injury, ALP and bilirubin are sensitive markers to investigate about biliary function, while albumin reflects liver synthetic function [29]. Measurements were done according to the corresponding kits instructions using a colorimetric autoanalyzer [30].

### Measurement of antioxidants

#### Measurement of total antioxidant status (TAS)

Blood samples were centrifuged at 4000 rpm for 10 min. Plasma TAS was measured using an automated colorimetric of the total antioxidant response (TAR) which was described previously [31]. In this method, Fe^2+^–o-dianisidine reacts with hydrogen peroxide by the Fenton reaction to produce OH^•^. The hydroxyl radical then reacts with o-dianisidine to produce yellow-brown colored dianisyl product. The color formation is increased with further oxidation reactions. By adding the samples, color formation is decreased by the antioxidant components of the plasma.

#### Measurements of liver superoxide dismutase (SOD)

The dissected tissues were immediately frozen in liquid nitrogen and stored at −70 °C. Later the tissues were homogenized in 1.15 % KCl solution and 20 % (W/V) homogenate, which was later, centrifuged and then used to measure SOD activity using spectrophotometric method. The method was based on the inhibition of a superoxide-induced nicotinamide adenine dinucleotide (NADH) oxidation by a chemical reaction in the presence of EDTA, manganese (II) chloride and mercaptoethanol as previously described [32].

#### Liver glutathione peroxidase (GSH-Px) activity

GSH-Px activity was determined in liver tissues [33]. Briefly this measurement is based on the conversion between the oxidized glutathione (NADPH) to the reduced form (NADP). The rate of absorbance during this conversion was measured using spectrophotometer at 340 nm for 3 min. The activity of GSH peroxidase was expressed as nmol of NADPH oxidized per min per mg DNA.

#### Liver catalase (CAT) activities

The method was previously described by Claiborne group [34]. Briefly 50 μL of liver tissue lysate was added to of H_2_O_2_ (10 mM, 0.95 mL) and later to reaction buffer (60 mM phosphate buffer; pH 7.0). Catalase activity was measured as micromoles of H_2_O_2_ consumed per minute at 25 °C. The changes in absorbance were recorded at 240 nm using spectrophotometer.

#### Liver lipid peroxidation assay

Oxidative stress was evaluated using Malondialdehyde (MDA) equivalents which were assessed utilizing the thiobarbituric acid (TBARS) as reactive substrate [35]. The colorimetric reaction between MDA and TBARS) were assayed (pH 2–3, 90 °C) for 15 min. The maximum absorption was recorded at 532 nm.

### Histopathological analysis

Liver tissue samples were taken from each animal, fixed in 10 % neutral formalin and after histological process of dehydration, tissue embedded in paraffin, section with a microtome set at a thickness of 5–6 μm. The tissue sections were stained with hematoxylin and eosin (H&E) for histopathological analysis and examined with a light microscope. The degree of liver tissue injury was evaluated semiquantitatively according to the method reported by Jamshidzadeh *et al*. [36]. The stained 5 μm sections were graded as follows: 0, absent; 1, minimal; 2, mild; 3, modest; 4, severe. The histological changes were evaluated in nonconsecutive, randomly chosen × 200 histological fields using light microscope, NIKON ECLIPSE E200. Neutrophils were counted in 50 high-power fields (×400). The pathologist performing the histologic evaluation had no knowledge about the treatment of the animals, from which the samples originated.

### Data analysis

The Statistical Package for Social Sciences (SPSS Inc., Chicago, IL, USA), version 17.0, was used for statistical analysis. All data are presented as mean ± SD. Before statistical analysis, all variables were checked for normality and homogeneity of variance by using the Kolmogorov-Smirnoff and Levene tests, respectively. The data obtained were tested by ANOVA followed by Tukey’s posthoc multiple comparison test. The Kruskal-Wallis test, followed by Mann–Whitney U posttest, was used for the analysis of degree of histopathological liver injury. *P* < 0.05 was considered statistically significant.

## Results

### Effect of turmeric on antioxidant status in rat liver

The liver antioxidant markers (SOD, GSH-Px and CAT) are shown in Table 2 for different groups. MTX significantly (*P* < 0.05) decreased SOD, GSH-Px and CAT activities in liver tissue compared to control, turmeric (100 mg/kg) and turmeric (200 mg/kg) groups. However, the groups, which were treated with MTX in presence of TUR, (200 mg/kg) showed significant increase in SOD, GSH-Px and CAT activity in liver tissue compared to MTX group (*P* < 0.05). On the other hand turmeric (100 mg/kg) and turmeric (200 mg/kg) control groups did not show any significant changes in the activities of SOD and GSH-Px, while CAT level were significantly increased compared to control group in the liver tissue (*P* < 0.05).

**Table 2:** Effect of turmeric on antioxidant status in rat liver

Groups	SOD (U/mg protein)	GSH-Px (U/mg protein)	CAT (U/mg protein)
Control	3.34 ± 0.32	3.9 ± 0.39	2.21 ± 0.24
TUR (100mgkg^−1^)	3.77 ± 0.39	4.22 ± 0.72	2.54 ± 0.51
TUR (200mgkg^−1^)	4.43 ± 0.53	4.67 ± 0.58	3.33 ± 0.65^a^
MTX (20mgkg^−1^)	1.76 ± 0.25^a^	2.12 ± 0.23^a^	1.03 ± 0.27^a^
TUR (100mgkg^−1^) + MTX	2.13 ± 0.41^a^	2.84 ± 0.26^a^	1.36 ± 0.14^a^
TUR (200mgkg^−1^) + MTX	3.08 ± 0.36^b^	3.23 ± 0.31^b^	2.11 ± 0.19^b^

Values of SOD, GSH-Px and CAT are shown as mean ± S.D.

*TUR* Turmeric, *MTX* Methotrexate, *SOD* superoxide dismutase, *GSH-Px* Glutathione peroxidase, *CAT* Catalase. ^a^Significant different with control group at *P* < 0.05. ^b^Significant different with MTX at *P* < 0.05

### Effect of turmeric on lipid proxidation and total antioxidant status in liver and plasma

The hallmarks of lipid peroxidation and total antioxidant (MDA and TAS) are shown in Table 3. Liver’s MDA content was significantly increased in MTX group compared to control group *(P <* 0.05). Whereas, the concentration of MDA in liver tissue was significantly decreased in group *VI* treated with turmeric 200 and MTX when compared to MTX group (*P* < 0.05). TAS was significantly decreased in MTX group while turmeric 200 mg/kg induce an increase in plasma TAS compared to control group (*P* < 0.05). In the group which TUR 200 and MTX was used, plasma TAS was significantly elevated compared to MTX group (*P* < 0.05).

**Table 3:** Effect of turmeric on lipid proxidation and total antioxidant status in liver and plasma

Groups	MDA (nmol/mg protein)	TAS (mmol Trolox Eq/L)
Control	2.34 ± 0.49	1.5 ± 0.43
TUR (100mgkg^−1^)	2.12 ± 0.52	1.78 ± 0.26
TUR (200mgkg^−1^)	1.9 ± 0.33	2.39 ± 0.41^a^
MTX (20mgkg^−1^)	4.27 ± 0.73^a^	0.78 ± 0.06^a^
TUR (100mgkg^−1^) + MTX	3.76 ± 0.61	1.08 ± 0.16
TUR (200mgkg^−1^) + MTX	3 ± 0.59^b^	1.42 ± 0.23^b^

Values of MDA and TAS are shown as mean ± S.D.

*TUR* Turmeric, *MTX* Methotrexate, *MDA* Malondialdehyde, *TAS* Total Antioxidant Status. ^a^ Significant different with control group at *P* < 0.05. ^b^ Significant different with MTX at *P* < 0.05

### Effect of turmeric on the serum ALT, AST, ALP, Bilirubin, ALB and TP

The results of serum liver biochemical hallmarks are presented in Table 4. MTX treatment significantly increased serum ALT, AST, ALP activity and bilirubin concentration (*P* < 0.05) while induced a significant decrease in serum ALB and TP (*P* < 0.05) compared to control group. Also, the same trends were observed in comparison between the group, which was treated with turmeric 100 + MTX and the control group. The group, which was treated with turmeric + MTX, significant had lower ALT, AST, ALP serum activity, and bilirubin concentration compared to the MTX group (*P* < 0.05). Likewise, significant increases were observed in ALB and TP concentrations in turmeric 200 + MTX group, compared to MTX group (*P* < 0.05).

**Table 4:** Effect of turmeric on the serum levels of ALT, AST, ALP, Bilirubin, ALB and TP

Groups	ALT	AST	ALP	Bilirubin	ALB	TP
	(U/L)	(U/L)	(IU/L)	(mg/dl)	(g/dl)	(g/dl)
Control	36.53 ± 2.1	51 ± 2.57	186.59 ± 8.68	0.81 ± 0.04	3.67 ± 0.28	6.65 ± 0.55
TUR (100mgkg^−1^)	34.43 ± 0.18	50.59 ± 2.3	188.43 ± 8.54	0.83 ± 0.06	3.44 ± 0.42	6.82 ± 0.59
TUR (200mgkg^−1^)	33.9 ± 0.45	49.26 ± 0.36	184.99 ± 8.62	0.85 ± 0.05	3.42 ± 0.54	7.05 ± 0.69
MTX (20mgkg^−1^)	49.91 ± 2.11^a^	74.83 ± 3.89^a^	276.61 ± 10.56^a^	1.46 ± 0.1^a^	2.36 ± 0.37^a^	4.28 ± 0.41^a^
TUR (100mgkg^−1^) + MTX	42.42 ± 1.32^a^	63.48 ± 0.25^a^	226.78 ± 9.25^a^	1.21 ± 0.08^a^	2.9 ± 0.2^a^	5.48 ± 0.46^a^
TUR (200mgkg^−1^) + MTX	39.07 ± 0.4^b^	57.83 ± 0.31^b^	209.42 ± 8.7^b^	0.88 ± 0.07^b^	3.48 ± 0.38^b^	6.15 ± 0.57^b^

Values are shown as mean ± S.D.

*TUR* Turmeric, *MTX* Methotrexate, *ALT* Alanine Aminotransferase, *AST* Aspartate aminotransferase, *ALP* Alkaline phosphatase, *ALB* Albumin, *TP* Total protein. ^a^Significant different with control group at *P* < 0.05. ^b^Significant different with MTX at *P* < 0.05

### Effect of turmeric on liver to body weight ratio

**Fig. 1:**
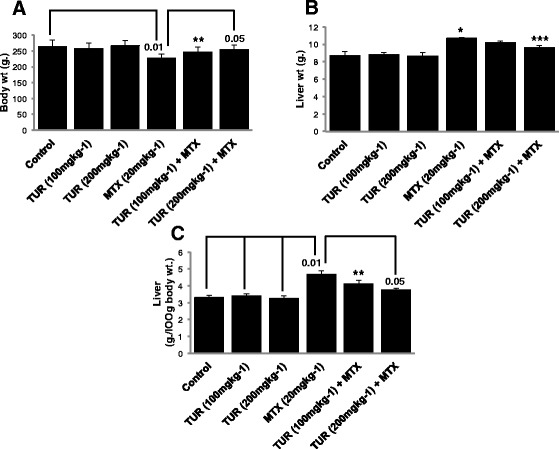
Effect of turmeric and methotrexate on body weight, liver weight, and liver weight/body weight ratio. **a** The body weight, (**b**) liver weight, and (**c**) body weight/liver weight ratio are shown. Values of body weight and liver weight are shown as mean ± S.D.* *P* < 0.01 compared with control, ** *P* < 0.05 compared with control, *** *P* < 0.05 compared with MTX group

The liver/body weight ratios in different groups are shown in Fig. 1. The body weight loss was observed for rats receiving MTX (*P* < 0.01) and MTX + turmeric 100 mg/kg (group *V*) (*P* < 0.05) compared to control (Fig. 1a). The body weight loss in-group treated with MTX + turmeric 200 mg/kg was also observed, as compared with the control group. However, the body weight loss was not significant (*P* > 0.05) in this group. The liver weights of groups *IV*, *V*, *VI* were significantly higher than the control group, suggesting hypertrophy of the liver (Fig. 1b). This is further emphasized by the fact that liver weight/body weight ratio of group *IV* was significantly (*P* < 0.01) higher than the groups *I*, *II*, and *III* (Fig. 1c)*.* In addition, a significant decrease in liver to body weight ratio was observed in animals treated with MTX + turmeric 200 mg/kg compared to group receiving MTX (*P* < 0.05). The liver/body weight ratio was higher in the group given MTX + turmeric 100 mg/kg in comparison with the control group (*P* < 0.05) (Fig. 1c).

### Histopathology study

**Fig. 2:**
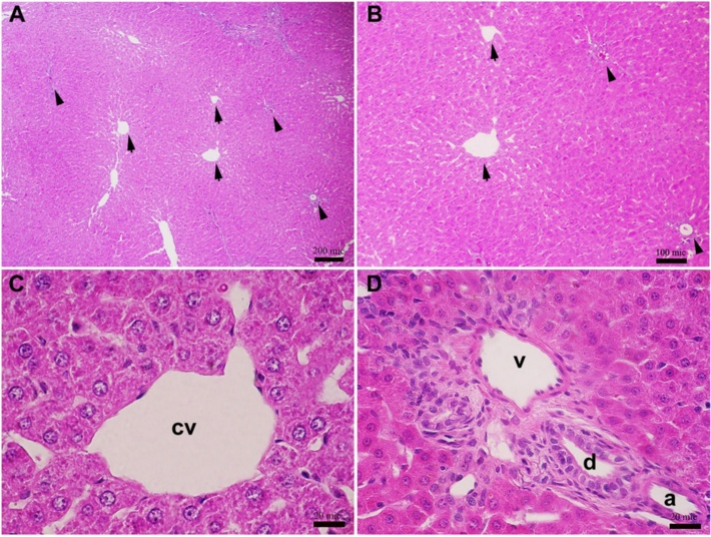
Histopathology examination of liver sections of the control group. **a**, **b** Microscopic view from the liver tissue of a rat belonged to healthy/control group stained with H&E (group *I*). **c** Microscopic view from the pericentral zone of rat liver stained with H&E (group *I*). **d** Microscopic view from the periportal zone of rat liver stained with H&E. Portal triad structures comprise of portal vein (v), hepatic artery (a), and bile duct (d) (group *I*). (central vein = cv), Scale bars = 200 μm in A; 100 μm in B; 20 μm in C and D

Histopathology examination of liver sections of the normal group were stained with H&E, showed in low magnification with normal appearance of central vein (cv, indicated by arrow) and portal triad structures (arrowhead) (Fig. 2ab). The cord-like structure of distinct polyhedral-shaped large hepatocytes well-preserved cytoplasm and rounded euchromatic nucleus with prominent nuclei showed around the central vein (pericentral zone) (Fig. 2c). The normal appearance of portal vein (v), artery (a), and bile duct (d) showed in portal triad that embedded in hepatocytes cords in periportal zone (Fig. 2d).

**Fig. 3:**
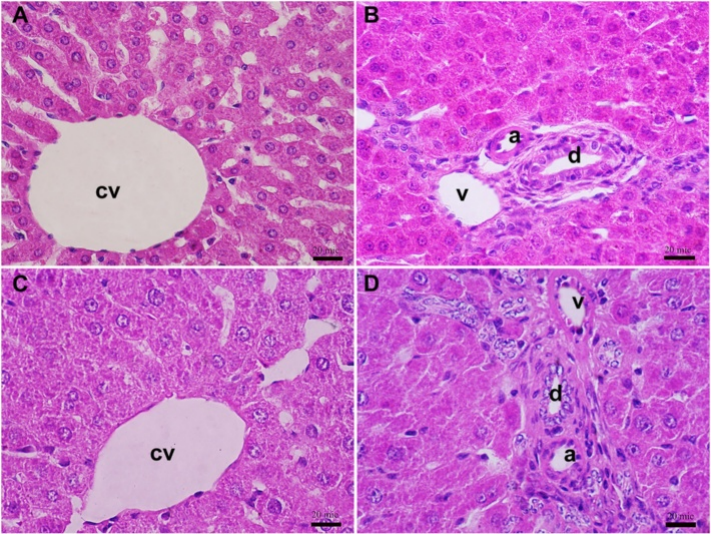
Histopathology examination of liver sections of the group treated with tumeric. **a** Microscopic view from the pericentral zone of a rat liver belonged to turmeric (100 mg/kg) treatment group stained with H&E (groups *II* and *III*). **b** Microscopic view from the periportal zone of a rat liver belonged to turmeric (100 mg/kg) treatment group stained with H&E. **c** Microscopic view from the pericentral zone of a rat liver belonged to turmeric (200 mg/kg) treatment group stained with H&E (groups *II* and *III*). **d** Microscopic view from the periportal zone of a rat liver belonged to turmeric (200 mg/kg) treatment group stained with H&E (groups *II* and *III*). Portal triad structures comprise of portal vein (v), hepatic artery (a), and bile duct (d) (central vein = cv, Scale bars = 20 μm in A, B, C and D)

In turmeric (100 mg/kg) treatment group, no obvious histopathological changes were observed in the liver section of this group in pericentral zone (Fig. 3a) and periportal zone (Fig. 3b). In examination of hepatic tissue from turmeric (200 mg/kg) treatment group, liver tissue had normal appearance and there were no histopathological changes as shown in pericentral zone (Fig. 3c) and periportal zone (Fig. 3d).

**Fig. 4:**
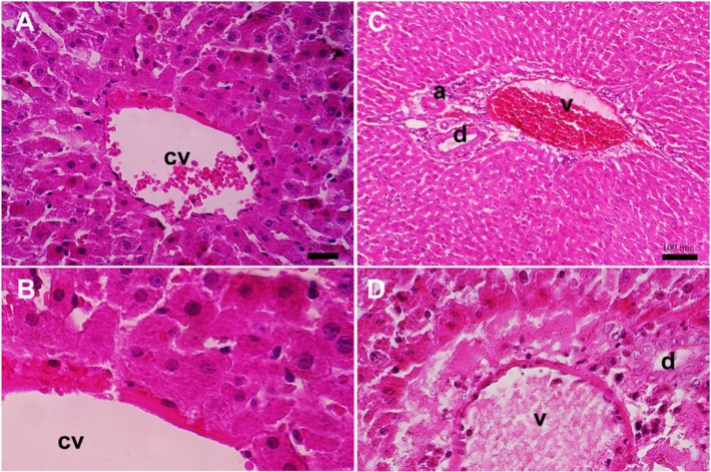
Histopathology examination of liver sections of the group treated with MTX. **a**, **b** Microscopic view from the pericental zone rat liver belonged to MTX-group stained with H&E (group *IV*). **c**, **d** Microscopic view from the periportal zone of rat liver belonged to MTX-group stained with H&E. Portal triad structures comprise of portal vein (v), hepatic artery (a), and bile duct (d) (group *IV*) (central vein = cv). Scale bars = 20 μm in A and D; 100 μm in B

Examination of liver tissues from MTX treatment group showed severe histopathological changes that prominent around dilation and congestion of central vein (cv) and portal vein (v) (Fig. 4). In pericentral zone of liver tissues from MTX treatment group showed inflammation and degenerating hepatocytes with condense nuclei (Fig. 4ab). The severe histopathological changes were prominent in periportal zone with appearance of hyperemia of portal vein, and inflammatory cells infiltration (Fig. 4cd). The necrotic cells and degenerating hepatocytes with condense nuclei and lack of polygonal-shaped outline were obvious in periportal zone that indicate severe toxicity effects of MTX to liver and hepatocyte (Fig. 4d).

**Fig. 5:**
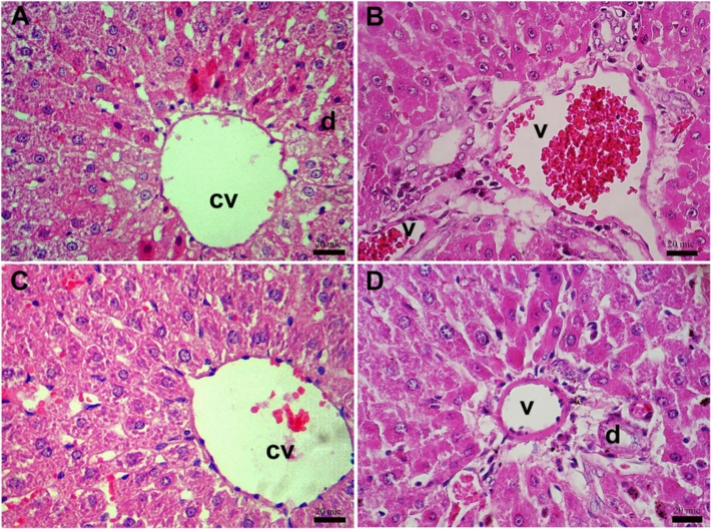
Histopathology examination of liver sections of the group treated with tumeric and MTX. **a** Microscopic view from the pericentral zone of a rat liver belonged to turmeric (100 mg/kg) + MTX treatment group stained with H&E (groups *V* and *VI*). **b** Microscopic view from the periportal zone of a rat liver belonged to turmeric (100 mg/kg) + MTX treatment group stained witch H&E (groups *V* and *VI*). **c** Microscopic view from the pericentral zone of a rat liver belonged to turmeric (200 mg/kg) + MTX treatment group stained with H&E (groups *V* and *VI*). **d** Microscopic view from the periportal zone of a rat liver belonged to turmeric (200 mg/kg) + MTX treatment group stained with H&E (groups *V* and *VI*). Portal triad structures comprise of portal vein (v), hepatic artery (a), and bile duct (d) (central vein = cv). Scale bars = 20 μm in A, B, C and D

**Fig. 6:**
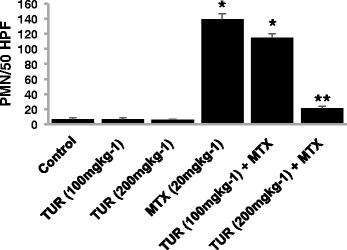
Effect of turmeric on PMN content of MTX-induced hepatic injuries. The number of neutrophils was obtained by counting these cells in 50 high-power fields. In control livers, relatively few neutrophils were found at sites of erosion. Similar results were obtained in group II and III. There was a significant increase in the number of neutrophils in the liver of rats treated with MTX (*P* < 0.05). There was no statistically significant difference between number of neutrophils in rats treated with MTX + turmeric (100 mg/kg) as compared to livers obtained from rats injected with MTX (group IV). However, the number of neutrophils accumulating in the liver in group (MTX + turmeric 200 mg/kg) has been significantly different compared to MTX treatment group (*P* < 0.05). Values are shown as mean ± S.D. * *P* < 0.05 compared with control, ** *P* < 0.05 compared with MTX group

To determine whether pretreatment of turmeric reduces the toxicity of MTX, we have treated animals with turmeric (100 and 200 mg/kg) and followed by administration of MTX. The histopathological studies of liver section from showed an improvement in damages caused by MTX to the hepatic tissues in a dose dependent manner (Fig. 5). Microscopic image from liver tissues of animals which have been treated with MTX in the presence of turmeric (100 mg/kg) (group *IV*) showed moderate pericentral zone hepatocytes degeneration with clear nuclei and nucleoli (Fig. 5a). A section from portal triad area showed that periportal zone has a moderate degeneration while mild hyperemia was still retained in portal vein (Fig. 5b). MTX treatment in presence of turmeric (200 mg/kg) treatment (group *VI*), showed degeneration significantly decreased in pericentral zone (Fig. 5c) and Periportal zone (Fig. 5d). Histopathological examination of livers form group *VI* shows that most of hepatocytes appeared with concentric arrangement and prominent nuclei and nucleoli. Quantitative assessment of the samples shown in Fig. 6 (microscopic evaluation) are presented in Table 5.

**Table 5:** Effect of turmeric on hepatic injuries

Groups	Degree of liver tissue injury	The Kruskal-Wallis tes
Control	0.0 ± 0.0	*P* < 0.001
TUR (100mgkg^−1^)	0.0 ± 0.0	
TUR (200mgkg^−1^)	0.0 ± 0.0	
MTX (20mgkg^−1^)	2.83 ± 0.78^a^	
TUR (100mgkg^−1^) + MTX	2.20 ± 0.61^a^	
TUR (200mgkg^−1^) + MTX	0.45 ± 0.18^b^	

Values are shown as mean ± S.D.

*TUR* Turmeric, *MTX* Methotrexate

0 = without injury, 1 = minimum injury, 2 = mild injury, 3 = moderate injury, 4 = sever injury. ^a^Significant different with control group at *P* < 0.05. ^b^Significant different with MTX at *P* < 0.05

We have next assessed the liver infiltration by neutrophils. In control livers, relatively few neutrophils were observed in sinusoids (7 ± 2 PMN/50 HPF). The turmeric treatment alone (100 and 200 mg/kg) had no significant effect on PMN level in the treated groups (7 ± 1 & 6 ± 1 PMN/50 HPF). The number of neutrophils increased significantly (*P* < 0.05) in MTX treated groups (237 ± 8 PMN/50 HPF). In general, turmeric at 100 mg/kg, had no significant effect on hepatic neutrophile counts (210 ± 5 PMN/50 HPF) regardless of the group. In contrast, turmeric at the dose of 200 mg/kg (21 ± 3 PMN/50 HPF) had significant effect (*P* < 0.05) when compared between MTX treated groups (Fig. 6).

## Discussion

**Fig. 7:**
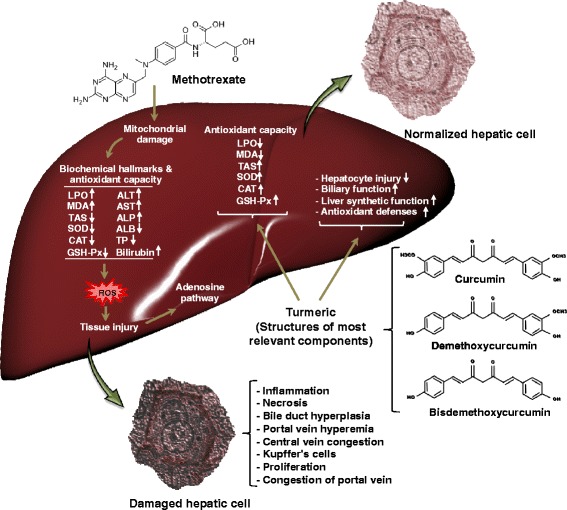
Schematic representation of the beneficial effects of turmeric on MTX induced liver toxicity. Turmeric plays an important role in modification of liver injury caused by MTX. The turmeric mostly exerts its effects by regulation of antioxidant capacity including enzymatic (SOD and CAT) and non-enzymatic antioxidants (GSH) as well as lipid peroxidation. It also modulates some liver related biochemical parameters causing in improvement of biliary and hepatic synthetic functions. Interestingly turmeric can normalize the histological changes in hepatocytes induced by MTX such as decrease in periportal degeneration, hyperemia, necrosis, and prevention of inflammatory cells infiltration

Several histopathology and serology markers have been previously used to investigate MTX-induced liver toxicity, including increased relative fatty acid changes in hepatocytes and sinusoidal lining cells, necrosis, inflammation and elevated activities of serum ALT and AST [13, 37, 38]. In the current study, histology outcomes confirm MTX-induced hepatotoxicity, and biochemical results are in agreement with the histological findings. As MTX mechanism of action is involved in folate antagonism, some of its toxicity effects such as anemia, neutropenia and stomatitis could be prevented or alleviated by folate supplementation. But there are other effects, which are unrelated to suppression of folate metabolism, including hepatic fibrosis, pulmonary fibrosis, nodulosis, lethargy and renal insufficiency [39–41]. Several studies have shown that MTX-induced hepatic fibrosis is mediated through oxidation and adenosine pathways (Fig. 7). Adenosine is a potent endogenous regulator of inflammation and tissue repair. Adenosine, which might be released from injured and hypoxic tissue in response to toxins and medications, may induce hepatic fibrosis in mice, presumably via adenosine receptor [42–44].

Our results showed prominent pathological changes in the liver tissue of MTX-treated animals including inflammatory cells infiltration, severe centrilobular/intermediate zone/ periportal degeneration, bile duct hyperplasia, hyperemia and necrosis observed compared to control and turmeric groups. Interestingly turmeric ameliorated the histological alteration in hepatocytes induced by MTX including decrease in periportal degeneration, hyperemia, necrosis, and prevention of inflammatory cells infiltration. The histology results were later confirmed by biochemical findings as have been mentioned in the previous sections.

Curcumin is the prevalent ingredient of turmeric and has been shown to possess several pleiotropic beneficial effects in many iatrogenic organ maladies [45, 46]. Animal studies have shown that curcumin might have positive effect against a wide range of human diseases, including diabetes, obesity, neurologic and psychiatric disorders, and cancer, as well as chronic illnesses affecting the eyes, lungs, liver, kidneys, and gastrointestinal and cardiovascular systems [47–49]. Curcumin has anti-inflammatory, anti-oxidant, pro-apoptotic, chemopreventive, chemotherapeutic, anti-proliferative, wound healing, anti-nociceptive, anti-parasitic, and anti-malarial effects as well [50–52]. However, besides curcumin, Tumeric extracts cointain many other active constituents such as turmerin, demethoxycurcumin, bisdemethoxycurcumin (two main active components of curcuminoids), turmeric proteins content, fats, minerals, carbohydrates, and essential oils, which could affect different biological functions and target specific organs, which depends on their molecular targets [53, 54]. Studies, over the two past decades, on turmeric extracts have indicated that its activities favorably compare to pure curcumin [27]. For example, curcumin is less potent than turmeric in delaying STZ-induced cataract [53] and in reducing blood glucose levels in type 2 diabetic [55].

Oxidative stress-induced cell damage is a major factor in the pathogenesis of many acute and chronic diseases [56–59]. The cell damage is initiated by the reaction of free radicals with membrane lipids and polyunsaturated fatty acids [60]. The current study showed that a single dose of MTX (20 mg/kg) caused significant hepatotoxicity. The level of MDA in liver tissue was significantly increased in MTX-treated rats compared to control group, which suggested that the peroxidation reaction was higher than control group in MTX-treated animals and correlated with the previous research findings about MTX-induced hepatoxocity [7, 11, 61, 62]. Our findings suggest that turmeric ROS scavenging-induced protective response markedly decreased MDA concentration. Comparable antioxidant effects were reported by other researchers in pulmonary, neurological, metabolic, and cardiovascular diseases [27]. Turmeric pre-treatment (200 mg/kg) showed higher improvement and recovery of liver tissue in presence of lipid peroxidation inhibition (e.g. 30 %) compared to group treated with turmeric (100 mg/kg). Hemeida and colleagues have previously shown that curcumin post-injection could decrease MTX-induced MDA production by around 24 % [13].

Our study also indicate that MTX-induced liver injury activates neutrophiles and results in liver infiltration by PMN. Neutrophil accumulation in the liver occurred parallel to the development of hemorrhage and parenchymal cell injury. Infiltration of neutrophils of liver tissues was concomitant with the oxidant/antioxidant status and changes in MDA levels. Increased MDA levels in the rat-livers after MTX administration suggests activation of an inflammatory response. We observed increased MDA levels in the liver tissue, and this may indicate that neutrophil accumulation and lipid peroxidation contributes to MTX-induced liver injury. Likely, the activated neutrophils located in the area of inflammation, may convert hydroperoxides into free radicals, triggering lipid peroxidation and hepatocyte cell membrane damage.

In our study, SOD and CAT activities were also significantly decreased in MTX treated group compared to healthy control group. SOD and CAT are the main enzymes, which are involved in H_2_O_2_ and ROS scavenging [63]. SOD and CAT activities were increased in all groups treated with turmeric-MTX compared to groups, which received MTX alone. SOD activity was increased 27 % and 75 % in the groups, which were pre-treated with tumeric (100 mg/kg and 200 mg/kg) before MTX exposure respectively, while CAT activity were increased 32 % and 105 % respectively. Similar results, in different tissues, were reported by others [13, 64–66].

The present study also shows that MTX could reduce GSH, which indicates imbalance between antioxidants and oxidants in the liver. As GSH is one of the most important parts of antioxidant defense system [67], when MTX decreases the level of GSH, cellular redox state alters and cells could be more sensitive to reactive oxygen metabolites [68]. Such findings have been observed in other experimental systems [69]. One of the possible mechanisms related to GSH depletion is based on the decrease of NADPH availability due to MTX inhibitory effects on glucose 6 phosphate dehydrogenase [70]. Glutamine is necessary for glutathione production. A study indicated that intravenous glutamine supplementation could protects liver cells from MTX-induced oxidative damage due to increasing intercellular GSH level [71] as turmeric increased GSH level with unknown mechanism. Our results show that turmeric not only affected enzymatic cellular antioxidant machinery (SOD and CAT), but also non-enzymatic defense system (GSH). Preadministration of turmeric (100 and 200 mg/kg) in groups *V* and *VI* for 30 days can significantly restore the GSH level about 34 % and 54 % respectively compared to groups that received MTX alone.

Total antioxidant status (TAS) reflects the total effect of all antioxidants in plasma [72]. In our study, the level of TAS in rats treated with MTX, were significantly lower compared to healthy control group. Previous studies indicated decreased antioxidant capability and increased oxidative stresses provokes hepatic damage [42, 56, 73]. In groups *V* and *VI* (turmeric + MTX), serum TAS were elevated compared to group *III* (MTX treated animals), but statistically significant results were obtained when we used higher concentration of turmeric (200 mg/kg). We have previously shown that dietary turmeric can markedly enhance TAS level under physilogical conditions in rats [74].

In our study, MTX increased serum activities of ALT, AST, ALP and bilirubin serum concentration and decreased the concentrations of ALB and TP, which are hepatotoxicity and impaired liver function indicators [75, 76]. ALT and AST are the major critical enzymes in the biological processes [77, 78]. Reports have suggested that their levels increase in different hepatic injures such as hepatitis and cirrhosis induced by alcohol, drugs, viruses and also under oxidative stress [79]. Also there are not similar studies about ALP, ALB, and TP levels in MTX induced liver toxicity. Our investigation revealed that in turmeric 200 + MTX group, serum activities of ALT, AST, ALP and serum bilirubin concentration were decreased, and levels of ALB and TP were increased compared to MTX group which correlates to previous reports [13, 80]. The low and high concentrations of turmeric did not induce any significant changes in ALT, AST and ALP levels compared to control group which confirms the previous finding [81]. Recently Fu et al. demonstrated that turmeric could effectively protect liver against CCL4-induced injury. They also showed that it prevented AST and ALT augmentation and improved the liver cell function [76]. In a randomized clinical trial on patients with increased levels of ALT, fermented turmeric powder decreased serum ALT and AST while there were not any significant changes in serum ALP, TB and lipids concentrations [82]. The results are varying among the investigators because of different methods of study or different population, which has been studied.

## Conclusion

In conclusion turmeric could prevent MTX hepatotoxicity and it might have a great potential for application in future disease therapy strategies. Liver-, breast- and other cancers often require aggressive chemotherapy, preferably targeting cancer stem cells [83–89]. The future clinical trials should be arranged to investigate the actual effect of turmeric in human patients and the possibility of considering this medical herb as a potential additive to MTX anticancer drugs.

## Abbreviations

ALP: Albumin
ALP: Alkaline phosphatase
ALT: Alanine transaminase
ANOVA: Analysis of variance
AST: Aspartate Aminotransferase
BDH: Bile duct hyperplasia
CAT: Catalase
CV: Central vein
DMSO: Dimethyl sulfoxide
EDTA: Ethylene diamine tetracetic acid
GPx: Glutathione peroxidase
GSH: Glutathione
GSH-Px: Glutathione peroxidase
GST: Glutathione-S-transferrase
H&E: Hematoxylin and Eosin
HPF: High-power fields
ICI: Inflammatory cells infiltration
LPO: lipid proxidation
MDA: Malondialdehyde
MTX: Methotrexate
NADH: Nicotinamide adenine dinucleotide
PMN: Neutrophils
PV: Portal vein
ROS: Reactive oxygen species
SOD: Superoxide dismutase
TAR: Total antioxidant response
TAS: Total antioxidant status
TBA: 2-thiobarbituric acid
TBARS: Thiobarbituric acid reactive substances
TP: Total protein
